# Long-lasting household damage from Cyclone Idai increases malaria risk in rural western Mozambique

**DOI:** 10.1038/s41598-023-49200-3

**Published:** 2023-12-07

**Authors:** Kelly M. Searle, Dominique E. Earland, Albino Francisco Bibe, Anísio Novela, Vali Muhiro, João L. Ferrão

**Affiliations:** 1 University of Minnesota School of Public Health, Minneapolis, MN USA; 2 Escola Secondária de Sussundenga, Sussundenga, Manica Mozambique; 3 Direcção Distrital de Saúde de Sussundenga, Sussundenga, Manica Mozambique; 4 Consultores Associados de Manica, Sussundenga, Mozambique

**Keywords:** Climate sciences, Natural hazards, Health care, Medical research, Risk factors

## Abstract

Cyclone Idai in 2019 was one of the worst tropical cyclones recorded in the Southern Hemisphere. The storm caused catastrophic damage and led to a humanitarian crisis in Mozambique. The affected population suffered a cholera epidemic on top of housing and infrastructure damage and loss of life. The housing and infrastructure damage sustained during Cyclone Idai still has not been addressed in all affected communities. This is of grave concern because storm damage results in poor housing conditions which are known to increase the risk of malaria. Mozambique has the 4th highest malaria prevalence in sub-Saharan Africa and is struggling to control malaria in most of the country. We conducted a community-based cross-sectional survey in Sussundenga Village, Manica Province, Mozambique in December 2019-February 2020. We found that most participants (64%) lived in households that sustained damage during Cyclone Idai. The overall malaria prevalence was 31% measured by rapid diagnostic test (RDT). When controlling for confounding variables, the odds of malaria infection was nearly threefold higher in participants who lived in households damaged by Cyclone Idai nearly a year after the storm. This highlights the need for long-term disaster response to improve the efficiency and success of malaria control efforts.

## Introduction

Malaria represents an important global health problem and is concentrated primarily in sub-Saharan Africa^1^. *Plasmodium falciparum* parasites are the causative parasite that is associated with the most severe form of disease, which is the most prevalent species in this sub-Saharan Africa^1^. It is well documented that individual factors, primarily age, are associated with risk for *P. falciparum* infection and subsequent disease^1^. Those at younger ages with less immunity are at highest risk for morbidity and mortality related to infection, especially in the absence of high coverage of interventions or access to healthcare services^2^. However, more modifiable household and community risk factors are being explored as they could have a great impact on malaria control^3–10^.

Over the past several decades there has been increased awareness and attention towards malaria control, elimination, and eradication^1,11,12^. This increased awareness has been accompanied by increased funding for these efforts. The current interventions available to achieve such goals include but are not limited to: insecticide treated bedbet (ITN) distributions, indoor residual spraying (IRS) campaigns, case management through parasitological diagnosis and appropriate treatment with artemisinin combination therapy (ACT), and integrated community case management (iCCM) with community health workers (CHWs) conducting case management in areas with limited access to health facilities^1^. Many countries that increased coverage with these interventions have experienced marked declines in malaria incidence and prevalence as a result^11,12^. However there has been heterogeneities within and between countries in their distributions and responses to these interventions. One country with heterogeneous transmission and varied distribution and responses to current interventions is Mozambique^13^.

Currently, Mozambique has the 4th highest malaria cases and malaria mortality globally^1^. Malaria incidence increases from low in the southern region to high in the central and northern regions^13^. Elimination efforts are underway in the south which borders The Republic of South Africa and Eswatini^13^. The central and northern regions have had limited success with malaria control efforts. Manica Province in central Mozambique has the 4th highest prevalence of malaria out of the 11 provinces, and the highest in the central region of the country^13^.

For decades it has been hypothesized that human made climate change was quickly approaching and the transmission dynamics of infectious diseases would be impacted^14–21^. There has been specific interest in the impact of climate change on vector-borne diseases as they are intricately tied to environmental habitats where they interface with human populations^22–24^. Malaria has been a vector-borne disease of interest: for the following reasons: (1) malaria transmission is highly dependent on environmental and climate-related factors; (2) changing environmental conditions impact the effectiveness of global malaria control and elimination programs; and (3) the impacts of climate change are already apparent in many areas in the form of an increased frequency of severe weather events that negatively affect malaria control efforts by damaging and destroying housing and key infrastructure^25–28^.

The impacts of climate change are already apparent in Mozambique in the form of increased frequency of severe weather events^29–32^. Over the past 35 years, Mozambique has experienced over 75 declared natural disasters related to floods, droughts, and cyclones^33^. From 2005 to 2023 Mozambique had 16 named tropical cyclones and storms that severely impacted the population showing a trend from being infrequent anomalies to occurring annually^33^. The most notable of these was Cyclone Idai in 2019, which was one of the worst tropical cyclones recorded in the Southern Hemisphere^34^. The storm caused catastrophic damage and led to a humanitarian crisis in Mozambique^34,35^. In the aftermath, the affected population suffered a cholera epidemic on top of the housing and infrastructure destruction and loss of life^36,37^. The housing and infrastructure damage sustained during Cyclone Idai still has not been addressed in all affected communities, particularly those outside of the main impact area along the coast. This study quantifies this housing damage and the associated malaria risk. This associated malaria risk is of grave concern because storm damage results in poor housing conditions which are known to increase the risk of malaria^6,10,38^.

Our study was conducted in Sussundenga Village in Sussundenga District, Manica Province, Mozambique. Manica Province had an estimated malaria prevalence of 48% in 2018^13^. Sussundenga is a rural agrarian village located western Mozambique that sustained significant impacts of Cyclone Idai but remained outside of the major areas of response. Cyclone Idai made landfall in Beira, Sofala Province on March 14, 2019 and impacted Sussundenga as a tropical storm from March 15–March 17 with sustained rainfall and flooding sustained afterwards. The primary objective of this study was investigate and quantify the malaria risk associated with household damage caused by Cyclone Idai. This represents long-lasting damage that is not included in typical response measures but has the potential to increase malaria risk in the immediate and long-term aftermath and impact the efficiency of control efforts.

## Methods

### Study area

**Figure 1:**
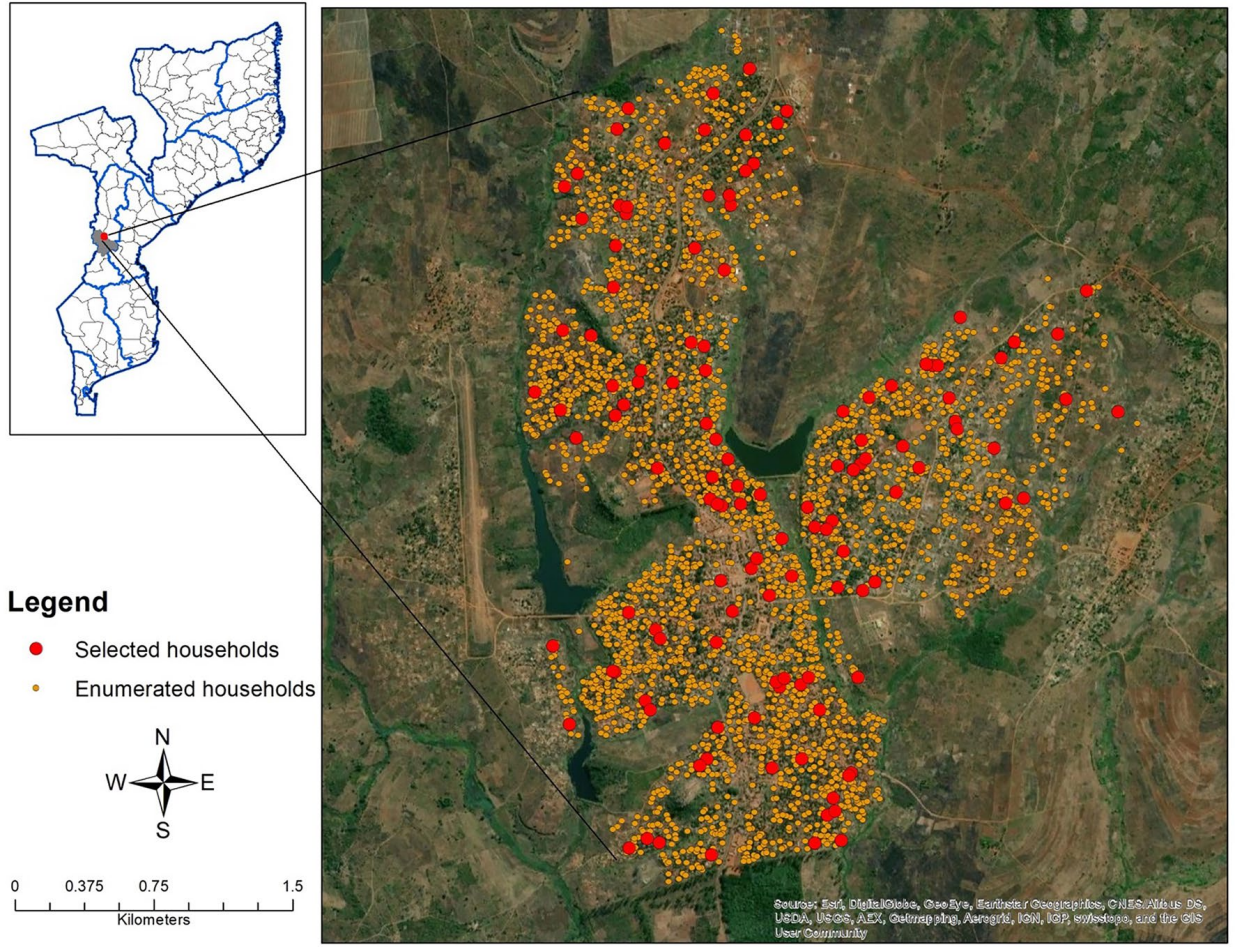
Map of enumerated households and selected households in Sussundenga, Mozambique.

The survey was conducted in Sussundenga village. Sussundenga village is approximately 42 km from the Provincial capital of Chimoio City in central Mozambique (Fig. 1). Sussundenga village, while a small village, is District capital of Sussundenga District and is a rural agrarian community with an approximate population of 19,112 residents^39,40^. From September to March, Manica Province has high temperatures and increased rainfall which contribute to the reported seasonally high malaria incidence. The main rural health center (RHC) in the village is the Sussundenga-Sede health center, with 14 smaller RHCs located in the district. The study sampled households from this village because of the high malaria incidence, rurality, impacts of Cyclone Idai, and accessibility to several RHCs for referral care. The primary malaria prevention intervention in the region at the time of the survey were insecticide treated nets (ITNs) for households distributed through the antenatal care center. This geographic area was selected for this study as it was both logistically feasible and experiences increased malaria incidence and prevalence compared to surrounding areas.

### Study design and data collection

A cross-sectional community-based survey was administered from December 2019 to February 2020 in Sussundenga village. This analysis focused on household level damage incurred during Cyclone Idai and malaria risk in the aftermath. Additional data regarding household structure, demographics, and use of malaria prevention were collected by data collectors as self-reported by participants. Satellite imagery was used to digitize and create a sampling frame with an enumeration list of 2889 structures representing households in Sussundenga village as the source population for a simple random sample. Households in this area are typically single structures or a group of structures where individuals in the same household use the same kitchen structure. A simple random sample method was used to select 125 households for screening with the goal of having 100 households that were inhabited and provided informed consent and for enrollment and to allow for potential misclassification of household structures and refusals. The screening process involved visiting the structure sampled from satellite imagery and determining if it was a household or misclassified non-household structure (e.g. small school, work building, shop). If it was a household structure, then the study team would invite the residents to enroll in the study. If residents were not home at the time of this visit or the household was not inhabited, the next structure on the list was visited. The number of households included for screening and enrollment was determined from estimated 5–6 total residents per household at the time of survey administration. This study was designed as a pilot study so the sample size was calculated to detect differences in specific risk factors (e.g. bednet use and healthcare access) between those with and without *P. falciparum* infection detected by rapid diagnostic test and collected additional information on housing structure and cyclone damage as other potential risk factors of interest. GPS coordinates were used to locate households for enrollment. Data collectors determined eligibility through a notification visit with the head of the household and assigned each household member a unique identifier.

Data collectors also obtained informed consent for all adult residents and parent/guardian permission for children between 3 months and 13 years old and assent for minors between 13 and 17 years old. Enrollment eligibility criteria was any full-time resident older than 3 months. After the notification visit, data collectors administered the electronic survey and recorded household GPS coordinates on a tablet computer using a REDCap® (Research Electronic Data Capture) mobile application. All participants present at the time of survey administration who were older than 13 years old completed the survey and parents provided responses for children 3 months old to 13 years old.

A study nurse collected a finger prick blood sample and administered a malaria rapid diagnostic test (RDT) [Right Sign Malaria P*f* (Biotest, Hangzhou Biotest Biotech Co, China]. All participants with positive results were referred to Sussundenga-Sede RHC for confirmation of diagnosis and treatment. As the study nurse and data collections team were affiliated with Sussundenga-Sede RHC these individuals were followed up to ensure they received proper care after the study visit. All data were collected and stored using the REDCap® server hosted at University of Minnesota School of Public Health and was treated confidentially^11,12^. Ethical review and approval for the study was completed by the Institutional Review Board (IRB) at the University of Minnesota [STUDY00007184] and from A Comissão Nacional de Bioética em Saúde (CNBS) at the Ministry of Health of Mozambique [IRB00002657] and was preformed within their guidelines and regulations. Informed consent was obtained from all adults and parental permission and assent were obtained from all minor children.

### Data analysis

All data analyses were preformed using Stata (StataCorp. Version 15.1). Household damage due to Cyclone Idai was collected as a categorical variable by self-report by the head of household as no damage, minor damage, significant damage, or destruction of the home. In events where the participants reported that their home was destroyed, the survey was conducted at the current residence of participants, which was the site of the destroyed household in the process of repair as no participants reported migrating due to the damage. This variable was collected by self-report, as data on repairs were also collected and when repairs (if any) were made. As such, data on damage and repairs were reported the same between households that did and did not have repairs. ITN use was collected as a binary variable of whether the participant slept under an ITN the night before or not. Household structure data was collected as the materials used to construct the floor, walls, and roof. Floor, walls, and roof structure were collected as categorical variables based on the type of material as natural, rudimentary, or modern. For floors, natural materials were soil or mud, rudimentary materials were bamboo, wood, or sticks, and modern materials were cement, brick, or tile. For walls, natural materials were straw or mud, rudimentary materials were mud blocks, sticks, or metal, modern materials were cement, fired brick, or treated wood. For roofs, natural materials were straw or leaves, rudimentary materials were bamboo, wood, or sticks, and modern materials were zinc, metal, or asbestos. For analyses these were recategorized as rudimentary and modern, with natural and rudimentary materials being aggregated into a single category. Windows were collected as a categorical variable of whether they were open or able to close partially or fully. This variable was analyzed as whether windows were open or able to close or not (either partially or fully). Household structure variables were collected by self-report with confirmation by direct observation by the data collectors. These variables also were the conditions of the household at the time of the survey, and not before the cyclone. Age in years and number of residents per household were analyzed as continuous variables.

Descriptive statistical analysis was conducted comparing the primary outcome variable (malaria infection by RDT) and primary exposure variable (household damage due to Cyclone Idai) by comparing proportions of those with and without malaria infection across the levels of household damage. Precision around these proportions was determined by 95% confidence intervals using Clopper–Pearson methods. Potential confounders were compared similarly across the levels of household damage, with proportions used for binary and categorical variables (ITN use, wall structure, roof structure, floor structure, windows closing, and head of household education level) and medians and interquartile ranges used for continuous variables (age and number of household residents).

Maps of the study area were created using ArcGIS Desktop version 10.6.1 [ESRI, Redlands, CA USA]. These maps include all enumerated and selected households, households with at least 1 positive RDT result and those with no positive RDT results, and household damage. These maps were created to visually explore the spatial distributions of selected households, the outcome of interest, and the primary exposure of interest.

Generalized estimating equations (GEE) logistic regression models were used to quantify the association between Cyclone Idai household damage and malaria risk. GEE models were chosen to account for household level correlation and non-independence of the primary exposure variable (household damage) and the shared environmental conditions related to the non-independence of the outcome variable (malaria infection). A univariable model of the association between Cyclone Idai household damage and malaria infection was built. Univariable models of the association between potential confounders (age, ITN use, wall structure, roof structure, floor structure, windows closing, head of household education level, and residents per household) were also constructed to quantify the association between these variables and malaria infection. As these variables were determined a priori as potential confounders, they were also included in the multivariable analysis regardless of the precision around the effect estimates.

A multivariable GEE logistic regression model was constructed to determine the relationship between Cyclone Idai household damage and malaria infection while adjusting for measured confounders. Odds ratios were used to determine the effect size of the association with 95% confidence intervals for the precision around these estimates.

## Results

**Figure 2:**
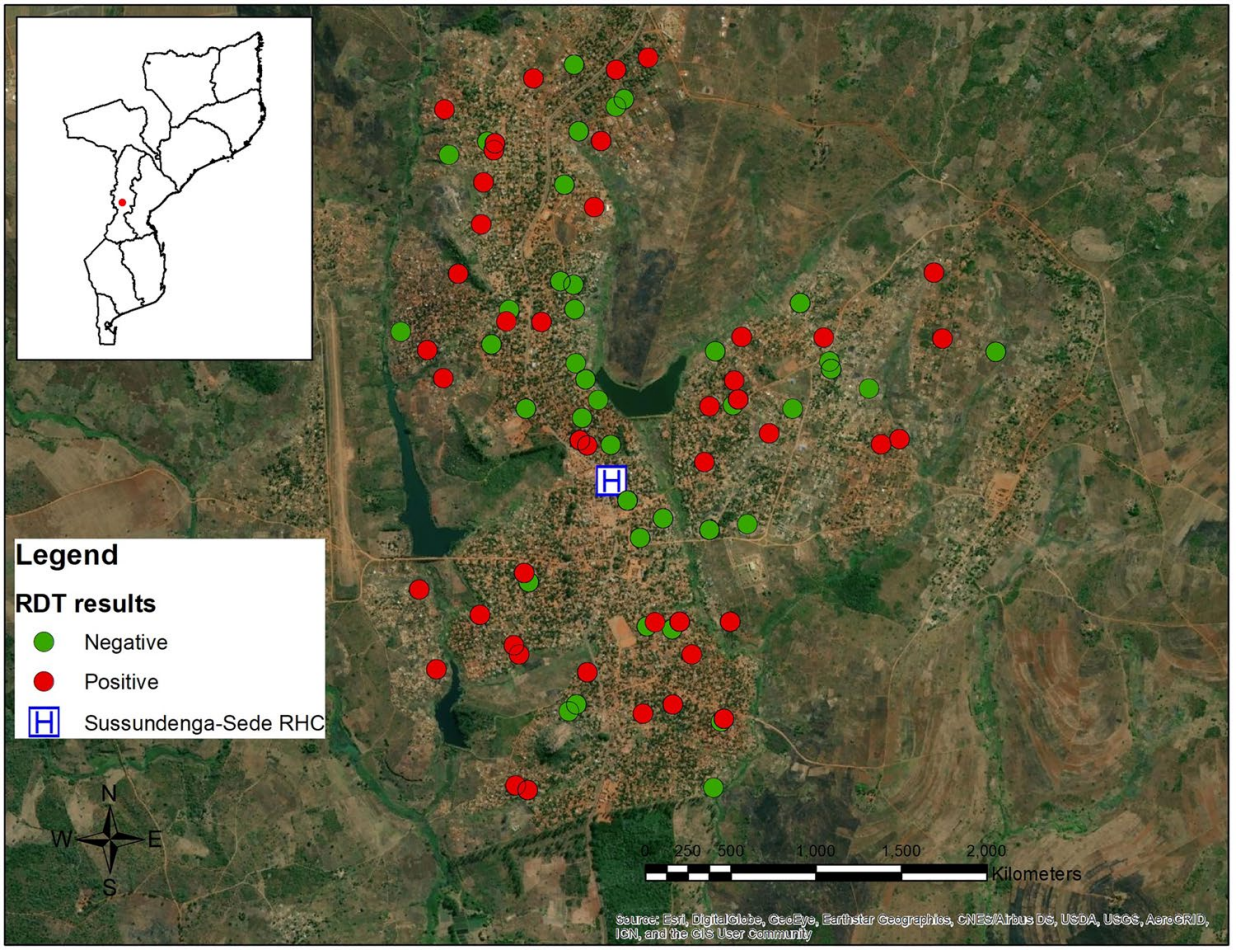
Distribution of households with RDT positive and negative participants.

**Figure 3:**
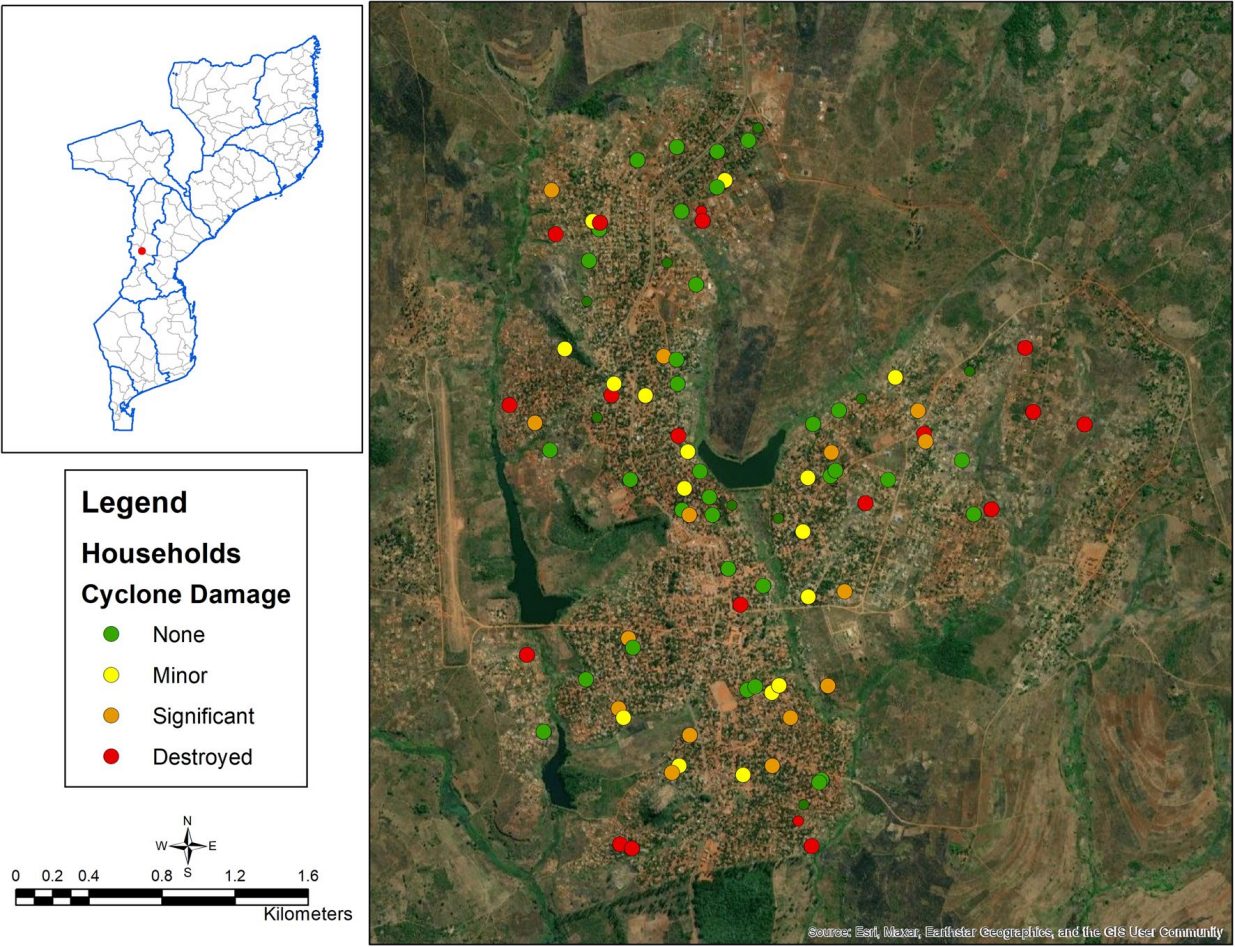
Distribution of household damage.

**Figure 4:**
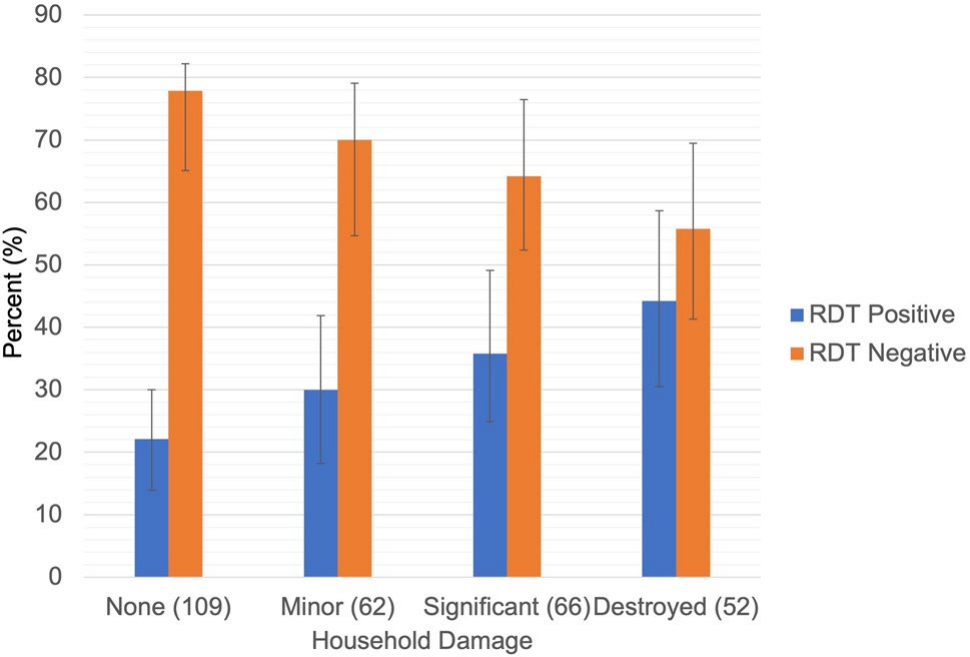
Malaria prevalence by level of household damage.

One hundred and twenty-five structures were visited, with 109 being individual households that were inhabited. These 109 households were approached to offer enrollment in the study with 84 households agreeing to participate. The study enrolled 289 participants from these 84 households who were available at the time of the study visit (Fig. 1). Overall, 59.3% of households and 62.4% of residents reported some level of household damage related to Cyclone Idai and the malaria prevalence measured by rapid diagnostic test (RDT) was 31.1%. There wasn’t clear clustering of either malaria positivity or household damage within the study sample in Sussundenga (Figs. 2, 3). Malaria prevalence increased with increasing levels of household damage related to Cyclone Idai (Table 1, Fig. 4). Malaria prevalence was higher in households with significant damage (35.8% [24.9–49.1]) or complete destruction (44.2% [30.5–58.7]) compared to households with no damage (22.1 [13.9–30.0]) (Table 1, Fig. 4). ITN use was also lower among individuals with any household damage, compared to those with none, but this lacked precision. ITN use was only 65.5%, and of those who reported not using an ITN 61.3% indicated that they did not own an ITN. Household structure was highly correlated with damage due to Cyclone Idai. Those with minor damage (43.6% [31.0–56.7]), significant damage (35.8% [24.9–49.1]), and destruction (46.9% [30.5–58.7]) had a lower prevalence of modern walls compared to those with no damage (91.7% [84.9–96.2]) (Table 1). This same trend was seen comparing flooring and closable windows (Table 1). Those with significant damage (84.1% [75.7–93.6]) or destruction (76.9% [63.2–87.5]) had a lower prevalence of modern roofs compare to those with no damage (96.3% [90.9–99.0]) (Table 1). Head of household education level was also strongly correlated with household damage due to Cyclone Idai. Those who lived in household with minor damage (25% [14.2–36.7]), significant damage (1.5% [0.0–8.2]), or destruction (19.2% [9.6–32.5]), were less likely to have completed secondary school or higher compared to those with no damage (56.0% [46.1–65.5]) (Table 1). There were no differences in age or number of residents per household between categories of household damage.

**Table 1:** Characteristics of the study population by level of household damage due to Cyclone Idai.

	Household damage							
	None (109)	95% CI or IQR	Minor (62)	95% CI or IQR	Significant (66)	95% CI or IQR	Destroyed (52)	95% CI or IQR
RDT results (% [N])								
Positive	22.1 [23]	[13.9–30.0]	30.0 [18]	[18.2–41.9]	35.8 [24]	[24.9–49.1]	44.2 [23]	[30.5–58.7]
Negative	77.9 [81]	[65.1–82.2]	70.0 [42]	[54.7–79.1]	64.2 [43]	[52.4–76.5]	55.8 [29]	[41.3–69.5]
Age (years) (median [IQR])	15	[7–26]	18	[10–27]	19	[11–31]	18	[12–27]
Residents (number) (median [IQR])	4	[3–6]	5	[3–10]	7	[5–7]	4	[3–6]
ITN use (% [N])								
Yes	76.2 (83)	[67.0–83.8]	61.3 (38)	[48.1–73.4]	69.7 (46)	[57.1–80.4]	44.2 (23)	[30.5–58.7]
No	23.8 (26)	[16.2–33.0]	38.7 (24)	[26.6–51.9]	30.3 (20)	[19.6–42.9]	55.8 (29)	[41.3–69.5]
Wall structure (% [N])								
Modern	91.7 [100]	[84.9–96.2]	43.6 [27]	[31.0–56.7]	35.8 [24]	[24.9–49.1]	46.9 [23]	[30.5–58.7]
Traditional	8.3 [9]	[3.8–15.1]	56.4 [35]	[43.3–69.0]	64.2 [43]	[52.4–76.5]	53.1 [26]	[35.8–64.2]
Roof structure (% [N])								
Modern	96.3 [105]	[90.9–99.0]	91.9 [57]	[82.2–97.3]	85.1 [57]	[75.7–93.6]	76.9 [40]	[63.2–87.5]
Traditional	3.7 [4]	[1.0–9.1]	8.1 [5]	[2.7–17.8]	14.9 [10]	[7.5–26.1]	23.1 [12]	[12.5–36.8]
Floor structure (% [N])								
Modern	78.9 [86]	[70.0–86.1]	50.0 [31]	[37.0–63.0]	43.3 [29]	[31.7–56.7]	19.2 [10]	[9.6–32.5]
Traditional	21.1 [23]	[13.9–30.0]	50.0 [31]	[37.0–63.0]	56.7 [38]	[44.8–69.7]	80.8 [42]	[67.5–90.4]
Windows (% [N])								
Closable	30.3 [33]	[21.8–39.8]	11.3 [7]	[4.7–21.9]	13.4 [9]	[6.4–24.3]	19.2 [10]	[9.6–32.5]
Open	69.7 [76]	[60.2–78.2]	88.7 [55]	[78.1–95.3]	86.6 [58]	[77.5–94.6]	80.8 [42]	[67.5–90.4]
Head of household education (% [N])								
None or primary only	25.7 [28]	[17.8–34.9]	53.3 [32]	[38.6–64.5]	52.2 [35]	[40.3–65.4]	75.0 [39]	[61.1–86.0]
Secondary first level	18.3 [20]	[11.6–26.9]	21.7 [13]	[11.7–33.2]	46.3 [31]	[34.6–59.7]	5.8 [3]	[1.2–15.9]
Secondary second level or higher	56.0 [61]	[46.1–65.5]	25.0 [15]	[14.2–36.7]	1.5 [1]	[0.0–8.2]	19.2 [10]	[9.6–32.5]

In the univariable models significant damage (OR: 1.97 [0.99–3.88]) and destruction (OR: 2.79 [1.36–5.72]) were associated with malaria infection compared to those who sustained no household damage (Table 2). Increased age was associated with decreased odds of malaria infection (OR: 0.97 [0.95–0.99]. Modern flooring was also associated with decreased odds of malaria infection (OR: 0.49 [0.30–0.81]) (Table 2). Other potential confounding variables were not found to be associated with malaria with precision in univariable models but were included in the multivariable model due to their association with household damage and a priori hypotheses and conceptual frameworks.

**Table 2:** Univariable and multivariable GEE logistic regression model results using RDT results as the outcome of interest.

	Unadjusted		Adjusted	
	OR	95% CI	OR	95% CI
Cyclone Damage				
None	REF		REF	
Minor	1.51	[0.73–3.10]	1.59	[0.64–3.93]
Significant	1.97	[0.99–3.88]	2.78	[1.07–7.24]
Destroyed	2.79	[1.36–5.72]	2.97	[1.18–7.44]
ITN use				
No	REF		REF	
Yes	0.62	[0.37–1.03]	0.83	[0.45–1.51]
Wall structure				
Traditional	REF		REF	
Modern	0.64	[0.39–1.06]	1.23	[0.49–3.11]
Roof structure				
Traditional	REF		REF	
Modern	0.49	[0.23–1.05]	0.6	[0.23–1.59]
Floor structure				
Traditional	REF		REF	
Modern	0.49	[0.30–0.81]	0.58	[0.22–1.54]
Windows				
Closable	REF		REF	
Open	1.24	[0.68–2.26]	2.09	[0.97–4.49]
Head of household education level				
None or primary only	REF		REF	
Secondary first level	0.66	[0.35–1.27]	0.82	[0.37–1.83]
Secondary second level or higher	0.61	[0.33–1.09]	1.29	[0.57–2.90]
Residents per household (number)	1.05	[0.97–1.14]	1.04	[0.93–1.16]
Age (years)	0.97	[0.95–0.99]	0.83	[0.94–0.98]

**Figure 5:**
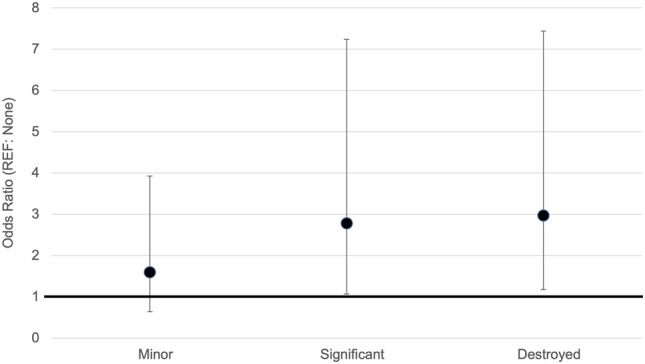
Adjusted odds ratios from multivariable logistic regression model of household damage and malaria infection.

After accounting for confounding variables, the association between damage incurred during Cyclone Idai and malaria risk was substantial. In the multivariable model significant household damage due to Cyclone Idai was associated with a 2.78 [95% CI 1.07–7.24] times increased odds of malaria infection compared to households with no damage (Table 2, Fig. 5). Destruction of the household due to Cyclone Idai was associated with a 2.97 [95% CI 1.18–7.44] times increased odds of malaria infection compared to household with no damage (Table 2, Fig. 5).

## Discussion

The impacts of climate change on malaria risk have previously been explored using simulations models and predictions based on expected changes in the environment and these impacts on the mosquito vectors^41–50^. However, climate change is already impacting malaria risk and the efficiency of control and elimination programs through increased frequency and severity of extreme weather events^51^. Our study demonstrates that observational epidemiologic studies can effectively quantify these associations, specifically the impacts of long-lasting housing damage on malaria risk in the aftermath of Cyclone Idai.

We found that participants who experienced household damage incurred during Cyclone Idai increased the odds of malaria infection by nearly threefold compared to those who did not experience household damage in an area of already high malaria incidence and prevalence while accounting for confounding factors. This is a substantial concern, considering our data collection was conducted nearly one year after Cyclone Idai occurred. Additionally, within our study population, only one household reported receiving any assistance from response organizations in the aftermath of Cyclone Idai.

Interestingly, outside of household damage due to Cyclone Idai only age held an association with malaria infection with precision in our fully adjusted model. The lack of an association between ITN use and malaria infection in our model stands out as it contrasts with consistent findings in many other studies^52–54^. We also failed to see associations between household construction materials and malaria infection in our adjusted model, which has been shown across different settings^3,4,6^. However, our analyses were focused specifically on the impacts of damage to the household which was likely also associated with the ITN use as well as household construction material. Therefore, it is likely that these variables in our model truly are confounding our main association and as such lack in statistical precision when looked at individually rather than as part of the larger model and conceptual framework. We assessed the correlation between construction materials of walls, roofs, and floors and found that they were statistically significantly correlated, which could have impacted the model. However, we included them separately in the model rather than a composite to determine the individual impact of each, as this is the level of practical significance and as it has been investigated in previous studies. Additionally, it should be noted that participants living in households with more rudimentary construction were more likely to sustain damage during the storm. This highlights a health inequity where more poor and marginalized members of communities are more likely to experience adverse events and impacts of severe storms.

While we found significant associations between household damage due to Cyclone Idai, there were limitations within our study. This was a cross-sectional study and therefore unable to make inferences about the change in household structure and damage directly from before and after the impacts of Cyclone Idai. Additionally, data on household damage came from self-report from the head of household. While Cyclone Idai was a major storm event, there is potential for recall bias in these responses that could not be confirmed by direct observation of the study staff during data collection. It should also be noted that asymptomatic infections are common in areas of high malaria transmission, which was the case in our setting. While we collected information on malaria symptoms, nearly all of the participants that were infected were asymptomatic making is difficult to include symptoms in our study for analysis. Finally, the sample size for this study was limited as a pilot study. The sample size was estimated to make comparisons between specific individual risk factors for *P. falciparum* infection, but not for household level risk factors.

As household construction has been highlighted as a risk factor for malaria, frequent and long-lasting damage to households due to severe storms will impact the efficiency of existing malaria control programs^4,6,8,10^. The National Malaria Control Program in Mozambique has experienced challenges in controlling malaria transmission outside of the southern region and the impacts of severe storms will exacerbate these challenges^13^. While immediate responses to severe storms have and should focus on injuries, life support, and cholera prevention and response, the long-term impacts should also become a priority^35,37,55^. Housing and infrastructure damage and malaria prevention should be included in this response to address the long-term impacts^1^.

## Data Availability

All data used for this study will be made available by request to the corresponding author.
